# Editorial: Innovative therapies against neurodegenerative disorders: from new active molecules to novel drug delivery systems

**DOI:** 10.3389/fphar.2025.1640758

**Published:** 2025-06-25

**Authors:** Panoraia I. Siafaka, Mehmet Evren Okur, Neslihan Üstündağ Okur

**Affiliations:** ^1^ Department of Life Sciences, School of Sciences, Program of Pharmacy, European University Cyprus, Nicosia, Cyprus; ^2^ Department of Pharmacology, Faculty of Pharmacy, University of Health Sciences, Istanbul, Türkiye; ^3^ Department of Pharmaceutical Technology, Faculty of Pharmacy, University of Health Sciences, Istanbul, Türkiye

**Keywords:** Alzheimer’s disease, neurodegenerative diseases, multiple sclerosis, nanoparticles, biomarkers, imaging

## Introduction

Neurodegenerative disorders as Huntington's disease (HD), Alzheimer's disease (AD), Parkinson's disease (PD), Multiple Sclerosis (MS)-Pietrantonio et al. are among the greatest and rather complex disorders for modern medicine (Siafaka et al., 2021a; Siafaka et al., 2022; Siafaka et al., 2021b; Derevyanko et al., 2025). These conditions involve the progressive loss of neuronal structure and function, affecting millions globally, thus imposing heavy social, familial, and economic burdens (Van Schependom and D’haeseleer, 2023).

For years, therapeutic options for these disorders have been very limited: at present, treatments only to alleviate symptoms, and no true options for modification of the disease process are available. However, in recent years, an unprecedented innovation surge has opened new possibilities. This Research Topic “Innovative Therapies Against Neurodegenerative Disorders: from New Active Molecules to Novel Drug Delivery Systems” comprises advanced studies and opinions reflecting the current situation toward a deeper understanding and more effective treatment of neurodegenerative diseases.

### From active molecules to precision medicine: redefining drug discovery

The essence of current breakthroughs is novel therapeutic agents that go beyond the conventional targets (Pietrantonio et al.). Disease-modifying therapies have started to be considered with monoclonal antibodies as aducanumab and lecanemab targeting amyloid-beta aggregates in Alzheimer’s disease. Those agents, however, are just the tip of the iceberg. Natural products (Polat et al., 2022), neurotrophic factors (Ibrahim et al., 2022), RNA-based therapies (Rabbani et al., 2025), and small-molecule modulators (McCrea et al., 2025) are all being explored, thus offering several strategies targeting diverse pathogenic mechanisms-from protein misfolding to mitochondrial dysfunction and neuroinflammation.

### Advancing drug delivery systems: an advancement of delivery systems

The blood-brain barrier (BBB) is one of the most challenging and complex barriers studied in neuropharmacology; it selectively protects the brain from pathogens and molecules but prevents drug penetration. Drug delivery systems can be functionalized being able to bypass this barrier. Engineered Nanoparticles (Jia et al), and functionalized nanoparticles loaded with nucleic acids-(Edith Muolokwu et al.), liposomes, and peptide-based vectors are developed to deliver therapeutic agents straight into the central nervous system, often with targeting ability.

Non-invasive techniques like focused ultrasound (FUS), on the other hand, allow temporary and localized disruption of the BBB, allowing much greater delivery efficiency without compromising safety (Mehta et al., 2024). The vectors of gene therapy, especially those of AAV (adeno-associated viruses), are also being repurposed to carry therapeutic genes directly to brain regions affected (Abulimiti et al., 2021), offering promise for the long-term correction of genetic and proteinopathies.

### Diagnostics and monitoring tools: toward early intervention

The efficient treatment demands an early and precise diagnosis; improvised techniques in imaging, fluid biomarkers (Cao et al., 2025), and nanotheranostics (Siafaka et al., 2021c) are continuously bringing into possibility the detection of neurodegenerative changes prior to the manifestation of clinical symptoms. These tools can be applied to observe the responses of treatments, furthering patient stratification, and guiding adaptive therapeutic strategies.

## Future directions

The manuscripts contributed to this Research Topic have made a case for a major shift in neurodegenerative disease research—moving from reactive treatment to proactive, personalized intervention. Even so, various challenges remain; large-scale production of novel therapies, high development costs, and the examination of larger populations over longer periods of time in clinical studies.

Collaborations across disciplines such as neuroscience, pharmacology, nanotechnology, genomics, etc are necessary. Regulatory bodies should closely monitor the innovations to ensure safe and timely translation from point of discovery to patient care.

## Conclusion

At present, neurodegenerative disease research is entering a transformative era. Molecules innovation, improved delivery systems, and diagnostic advances can be utilized for the management of these devastating disorders. We hope that the publications of this topic can contribute to present and future investigations for the management of neurodegenerative disorders. Scientists from various disciplines working together and continuing to innovate, can fulfill a world where the diagnosis of neurodegeneration no longer remains a death sentence but a manageable condition, and why not - a preventable one.
